# *In silico* Evolution and Comparative Genomic Analysis of IncX3 Plasmids Isolated From China Over Ten Years

**DOI:** 10.3389/fmicb.2021.725391

**Published:** 2021-12-03

**Authors:** Baomo Liu, Yingyi Guo, Ningjing Liu, Jiong Wang, Feifeng Li, Likang Yao, Chao Zhuo

**Affiliations:** ^1^State Key Laboratory of Respiratory Disease, The First Affiliated Hospital of Guangzhou Medical University, Guangzhou, China; ^2^Department of Respiratory Medicine, The First Affiliated Hospital of Sun Yat-sen University, Guangzhou, China

**Keywords:** IncX3 plasmid, *bla*_*NDM*_, carbapenem, *Escherichia coli*, genome

## Abstract

IncX3 plasmids are correlated with the dissemination and acquisition of carbapenem resistance in *Enterobacteriaceae* and have been prevalent in China over the last 10 years. Since the distribution characteristics of IncX3 plasmids across China as well as their evolutionary traits for 10 years remain unclear, here we conducted a retrospective literature review and *in silico* comparative analysis of IncX3 plasmids in publicly available IncX3 plasmid genomes. IncX3 plasmids distributed in 17 provinces or cities were extracted for analysis, which tend to be specifically associated with hospital-isolated *Escherichia coli* ST410 from phylogroup A. Although the backbones of IncX3 plasmids have remained highly conservative over the last 10 years, the *bla*_*NDM*_ resistance genetic contexts on these plasmids could fall into five subtypes, among which AR_N1_I has been identified in *Enterobacter cloacae174* chromosome and AR_N5_I was simultaneously located on IncF and IncA/C plasmids. This suggests that the *bla*_*NDM*_ resistance gene environment can spread between different plasmids, between different bacterial genera, or between strains and plasmids, highlighting that it is imperative to adopt more stringent infection control measures targeting IncX3 plasmid spread.

## Introduction

IncX3 plasmids were discovered 10 years ago, since Ho et al. (2012) first isolated the *bla*_*NDM*_/IncX3 plasmid pNDM-HN380 in China in 2011. IncX3 seems to be the most common plasmid-incompatible type for carrying *bla*_NDM_ in China and even the world (Ma et al., 2020). However, no previous research has investigated the distribution characteristics of IncX3 plasmids across China as well as their evolutionary traits over the last 10 years. A substantial number of plasmid whole genome sequences from China have been reported over the last few decades, which make it possible to conduct long-term and large-scale plasmid comparison and evolutionary analysis.

Therefore, the current study using published literature about IncX3 plasmids isolated from China and the IncX3 plasmid complete gene sequence deposited in NCBI was presented (i) to describe the prevalence of IncX3 plasmids across China over approximately 10 years, (ii) to identify the genetic context of IncX3 plasmids to further clarify the mechanisms related to antibiotic resistance gene transfer, and (iii) to explore the diversity of IncX3 plasmids and refine the IncX3 subgroup. This project provided an excellent opportunity to facilitate the understanding of the mechanisms of IncX3 plasmids’ high prevalence in China and the mechanism of the wide dissemination of carbapenem-resistant strains.

## Materials and Methods

### Search Strategy

We searched the PubMed database for published research of IncX3 separated in China before August 15, 2020. There was no language restriction. Keywords included (“China” and “IncX3”).

Article inclusion criteria: (1) the strain containing the IncX3 plasmid was isolated from China; and (2) the basic information of the strain can be queried in the article, including strain species, isolation dates, cities, and specimen sources.

Exclusion criteria: (1) the strains and plasmids were not isolated from China; (2) there was no bacterial species information of the plasmid host; and (3) all other basic information except the plasmid name is missing.

Referring to selected literature and GenBank online information, we collected the basic information of all IncX3 plasmids including specimen sources, isolation dates, cities, and MLST types (see section “Results” in detail).

### Plasmid Replicon Verification and Database Construction

We took the Rep sequence of the IncX3 plasmid deposited in the PlasmidFinder database as a reference and used BLASTN to verify the incompatibility group of the extracted plasmid sequence (homology >90%, coverage >90%). The hit rate of BLASTN was manually reviewed, and only published sequences were included in our IncX3 plasmid database.

### Antibiotic Resistance Gene Annotation

We used Mega-BLAST (*e* value ≤0.0001, identity ≥70%) against the ResFinder database to compare and annotate antibiotic resistance genes located in plasmids and then performed a manual inspection.

### Comparative Genomics Analysis of IncX3 Plasmids

The *bla*_*NDM*_/IncX3 plasmid pNDM-HN380 (GenBank accession number: JX104760.1) in *Klebsiella pneumoniae* strain HN380 was adopted as a reference plasmid for comparison. BLAST was used to compare all completely sequenced IncX3 plasmids with pNDM-HN380. We counted the number of hits in different regions of pNDM-HN380 to determine the conserved regions. Annotation of the complete gene sequence was performed using Prokka for all collected plasmids.

### Plasmid Backbone Recognition, Multiple Sequence Alignment, and Visualization

Mauve was used to perform multiple sequence alignment of plasmid DNA sequences to identify backbone genes. Plasmid backbone genes were defined as regions of plasmid DNA whose sequences were highly conserved in all aligned genomes.

The local blast + method was used for pairwise comparison to determine the plasmid group. Snippy software was applied to identify single-nucleotide polymorphism (SNP), insertion, or deletion changes between the reference plasmid pNDM-HN380 and other plasmids. Visualization of the comparison results was generated by Easyfig software, and the produced pictures were marked with gene names using Adobe Illustrator CC 2019 software.

## Results

### The Distribution and Host Characteristics of IncX3 Plasmids Reported in China

By searching “China and IncX3” in the PubMed database, the genome sequences and basic information of 84 IncX3 plasmids involving 60 documents were collected (see Supplementary Table 1 for all collected plasmid information).

From 2011 to 2021, a total of 84 non-duplicate collected IncX3 plasmids were reported in 13 provinces or cities across China, namely, Shandong, Jiangsu, Zhejiang, Guangdong, Shaanxi, Chongqing, Sichuan, Henan, Hong Kong, Beijing, Shanghai, Hunan, and Jiangxi. In this collection, the reported IncX3 plasmid-containing strains were isolated from clinical specimens (60.7%, 51/84, blood, urine, or sputum), livestock animals (17.9%, 15/84), life environment (such as sewage samples and surface of subway instruments), anal swab screening of admitted patients, and retail food meat.

The collected isolates carrying IncX3 plasmids were inspected further. The hosts of IncX3 plasmids involved 15 species. *Escherichia coli* (51.2%, 43/84), *K. pneumoniae* (23.8%, 20/84), and *Enterobacter cloacae* (7.1%, 6/84) were the most common hosts. The IncX3 plasmid was also sporadically distributed in 12 other species of *Enterobacteriaceae*, namely, *Citrobacter freundii*, *Salmonella Typhimurium*, *Cronobacter sakazakii*, *K. aerogenes*, *K. quasipneumoniae*, *Klebsiella oxytocin*, *Klebsiella oxytoca*, *Kluyvera cryocrescens*, *M. morganii*, *Proteus mirabilis*, *Raoultella planticola*, *Raoultella ornithinolytica*, and *Salmonella Lomita*.

A remarkable diversity of sequence types (STs) was identified among IncX3 plasmid-containing *E. coli* isolates. Among the 43 *E. coli* isolates, 17 different STs were noted. The preferred STs seemed to be ST48 (6/33), ST410 (4/33), ST156 (4/33), ST167 (3/33), ST1114 (2/33), ST10 (2/33), ST617 (2/33), and ST641 (2/33). The other *E. coli* isolates belonged to single diverse STs (including ST46, ST3835, ST224, ST-744, ST44, ST1236, ST448, ST3076, and ST8809). It is worth noting that all ST48 *E. coli* were isolated from animals and ST410s were from clinical patients and hospital environments.

We also obtained phylotype data of 16 *E. coli* strains in our collection. Phylotype data suggested that more than half (68.8%, 11/16) belonged to non-pathogenic phylogroup A. Phylogroup B included three strains, and the remaining two strains belong to group C. Phylogroup A strains have been isolated from various sources, including clinical specimens, animals, human colonization, and environment. Four types of *bla*_*NDM*_ variants (*bla*_NDM–1_, *bla*_NDM–5_, *bla*_NDM–7_, and *bla*_NDM–21_) were identified in IncX3 plasmid-carrying phylogroup A strains.

### Carbapenem Resistance Genes Carried on IncX3 Plasmids

All the collected IncX3 plasmids were positive for carbapenemase genes, among which 81 (96.4%) of them were NDM-positive and 3 (3.6%) were OXA181-positive. The IncX3 plasmid carried eight types of NDM variants, namely, NDM-1 (22.2%, 18/81), NDM-5 (67.9%, 55/81), NDM-7 (3/81), NDM-21 (1.2%, 1/81), NDM-13 (1.2%, 1/81), NDM-17 (1.2%, 1/81), NDM-19 (1.2%, 1/81), and NDM-20 (1.2%, 1/81). No bla_*KPC*_-carrying IncX3 plasmid was recorded in China.

Notably, *bla_*NDM–*5_* was the most prevalent carbapenemase gene in the IncX3 plasmids, followed by the *bla*_NDM–1_ subtype. The *bla_*NDM–*5_*-carrying IncX3 plasmids included plasmids from clinical specimens, livestock animals, life environment, anal swab screening of admitted patients, and retail food, indicating the wide dissemination of NDM-5-producing carbapenem-resistant isolates mediated by quick transfer of IncX3 plasmids. Interestingly, IncX3 plasmids containing the *bla*_NDM–1_ gene could be recovered from patient clinical specimens, rectal swabs, and hospital environments, which showed a preferred in-hospital spread for this gene subtype.

### Whole Genome Sequence Grouping of IncX3 Plasmids

Among the 84 plasmids collected, a total of 76 IncX3 plasmid complete genome sequences were finally obtained. The analysis using Mauve and BlastN software identified that all 76 plasmids could be divided into nine subgroups by comparing sequences, that is, pNDM-HN380 (group1), pNDM_MGR194 (group2), pOXA181_EC14828 (group3), MH10T (group4), pNDM-BJ03 (group5), pGZ2-NDM (group6), pWLK-NDM (group7), pNDM5-LDR (group8), and pNDM5-GZ04_A (group9).

Comparison of plasmid sequences revealed nine distinct types, mainly because of the variety of the genetic load region (Table 1). Researchers have referred to “the gene load region” as the fragments containing inserted sequences and different resistance genetic context (Ho et al., 2012). For example, for the first featured IncX3 plasmid pNDM-HN380, the gene load region was between the *resolvase* gene and *hns* gene, and involved *bla*_SHV_, *bla*_NDM_, and their surrounding mobile genetic elements (*IS26*, *Tn3*, and *tnpA*).

**TABLE 1:** IncX3 plasmid gene load regions.

Name	Carbapenemase	Size (bp)	Other resistance genes
pNDM-HN380	NDM-1	23,031	*bla*_*SHV–12*_
pNDM-BJ03	NDM-1	31,079	*bla*_*SHV–12*_
pWLK-NDM	NDM-1	45,279	*ampC*^f^*, tetA, tetR*
pMGR194	NDM-5	15,157	–
MH10T	NDM-5	12,803	–
pGZ2-NDM	NDM-5	39,545	*tmrB*^a^*, tap*^b^*, ant1*^c^*, emrE*^d^**,
pNDM5-LDR	NDM-5	19,975	–
pNDM5-GZ04_A	NDM-5	26,207	*mphA*^e^*, tap*
pOXA181_EC14828	OXA-181	15,371	*qnrS1*

*^a^Tunicamycin resistance protein, which is a nucleotide antibiotic produced by the actinomycete Streptomyces lysosuperficus. It can inhibit the reproduction of viruses with envelopes (glycoproteins). It can inhibit the formation of glycoprotein by inhibiting the transfer of N-acetylglucosamine-1-phosphate to monophosphate long alcohol.*

*^b^Scaini et al. (2019).*

*^c^Prabhu et al. (2017).*

*^d^Saleh et al. (2018).*

*^e^Fong et al. (2017).*

*^f^Cephalosporinase.*

The gene load region of the nine subgroups differed in resistance gene composition and mobile genetic elements. Most of the gene load regions (6/9) included resistance genes, more than just the carbapenemase gene as shown in Table 1.

### IncX3 Plasmid Backbone Region Was Highly Conserved

**FIGURE 1:**
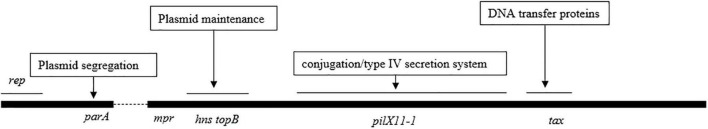
Plasmid backbone region map (dotted line indicates gene load region).

The gene organization of the IncX3 plasmid backbones was nearly identical to that of plasmid pNDM-HN380, and the counterparts shared >95% amino acid identities (Figure 1 and Supplementary Table 2). Among 75 plasmids compared with pNDM-HN380, only 21 plasmid backbones had a single-nucleotide change, or insertion/deletion of a single nucleotide, indicating that the IncX3 backbone has been highly conserved for 10 years.

### Subtype of *bla*_NDM_ Genetic Contexts Located in IncX3 Plasmids and Exploring the Mechanisms of Resistance Gene Transfer

**FIGURE 2:**
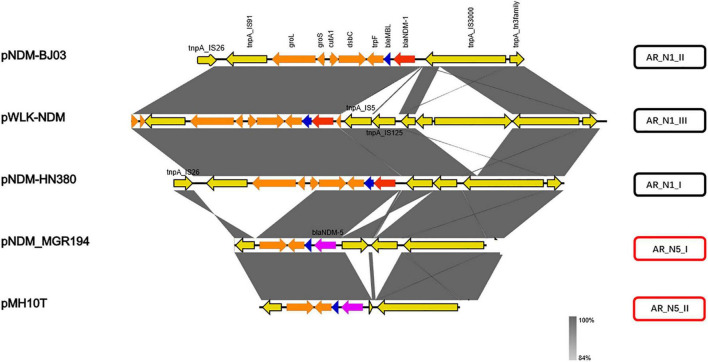
Summary of sub-classifications of resistance gene environment.

The *bla*_NDM_ genetic contexts identified fell into five groups according to the comparison analysis, including AR_N1_I–AR_N1_III and AR_N5_I–AR_N5_II (Figure 2). Among them, AR_N1 corresponds to *bla*_*NDM–*1_ genetic context and AR_N5 means *bla_*NDM–*5_* genetic context. Deletion or insertion of mobile gene elements (i.e., IS5 or IS125) and ORFs of unknown functions (i.e., *groL* and *groS*) accounted for the majority of the variations of these gene environments.

**FIGURE 3:**
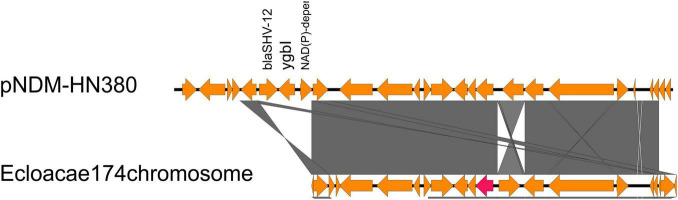
Comparison of AR_N1_I structure of pNDM-HN380 with the *Enterobacter cloacae 174* chromosome gene fragment in NCBI.

To detect the location of all *bla*_NDM_ genetic contexts other than IncX3 plasmids to discover the potential of transposon transfer, we searched all identified genetic context subtypes in the NCBI database. Surprisingly, we found that the genetic structure of AR_N1_I was nearly identical to a chromosome-encoded fragment containing *bla_*NDM–*1_* in *Enterobacter cloacae174* (accession number: CP020528, 1628321…1645166, Figure 3), whose gene composition of the chromosome-encoded fragment was flanked by *IS26*, in addition to holding AR_N1_I genetic context.

## Discussion

Previous studies have sporadically reported that the presence of IncX3 plasmids mediated the dissemination of carbapenem-resistant *Enterobacteriaceae* strains in China (Yang et al., 2020; Zhai et al., 2020; Zhou et al., 2020). However, no previous research has investigated the distribution characteristics of IncX3 plasmids across China as well as their evolutionary traits over the last 10 years. This study provided a comprehensive and updated prevalence and sequence characteristic profile of carbapenem-resistant IncX3 plasmids isolated from various sources in China. We have performed large-scale and long-term *in silico* analyses of reported IncX3 plasmids to elucidate IncX3 plasmid structural features over 10 years.

IncX3 plasmids are the most prevalent in *E. coli* and *K. pneumoniae* but sparse in other *Enterobacteriaceae*, where phylogroup A and ST410 *E. coli* isolated from patients seems to be the preferred host for IncX3 plasmids, highlighting that more infection control measures should be the target at these emerging specific associations between plasmids and bacterial clones. Consistently, Ma et al. (2020) demonstrated that IncX3 plasmids could often transfer to phylogroup A *E. coli* successfully and maintain high stability. The study of Huang et al. (2021) published in 2021 has found that *bla*_*NDM*_/IncX3 plasmids tend to be concentrated in ST410 *E. coli*, which also belongs to phylotype A. Our team also found a large number of ST410 *E. coli* with *bla*_NDM_-carrying IncX3 plasmids (data not shown).

The IncX3 plasmid carries at least eight kinds of *bla*_*NDM*_ gene variants, among which *bla_*NDM–*5_* is dominant. The *bla_*NDM–*5_*-carrying IncX3 plasmids were recovered from diverse sources, indicating the wide dissemination of NDM-5-producing carbapenem-resistant isolates mediated by quick transfer of IncX3 plasmids. However, *bla_*NDM–*1_*-carrying IncX3 plasmids were mainly distributed in the hospital, which show more limited dissemination compared with *bla*_NDM–5_.

We further divided carbapenem resistance genes and surrounding structural fragments obtained from IncX3 plasmid into five subtypes, AR_N1_I–AR_N1_III and AR_N5_I–AR_N5_II. The sub-classification of *bla*_*NDM*_ genetic contexts can help researchers understand the structural characteristics of resistance genes and explore its transmission mechanism. Our results found that AR_N1_I is highly homologous to *bla_*NDM–*1_* surrounding fragments encoded by *Enterobacter cloacae174* chromosome, indicating that *bla_*NDM–*1_* surrounding fragments could transfer between the IncX3 plasmid and *Enterobacter* chromosome. In 2020, Zou et al. (2020) reported that the genetic environment containing *bla_*NDM–*5_* could be divided into five subtypes. Among them, Type A corresponds to the AR_N5_I genetic environment in our study. The Type A genetic environment has been the most common (50%) genetic environment containing *bla*_*NDM*_, suggesting that the AR_N5_I genetic environment has an advantage in assisting the wide spread of *bla_*NDM–*5_*. Besides, the AR_N5_I genetic environment is also distributed on the IncF and IncA/C plasmids, and the IncA/C plasmid is a well-known broad host plasmid (Douarre et al., 2020), suggesting that the dissemination of *bla*_*NDM*_ gene will cross genus, not only limited to *Enterobacteriaceae* bacteria.

Therefore, the *bla_*NDM–*5_* genetic context carried by IncX3 plasmids can spread between different plasmids, between different bacterial genera, or between strains and plasmids. This suggests that controlling the spread of IncX3 plasmids has significant meaning for controlling the quick occurrence of carbapenem-resistant bacteria.

The genes on the backbone are responsible for maintaining the basic functions of the plasmid, including conjugation transfer and plasmid progeny distribution. The comparative analysis of the plasmid backbone region shows that the IncX3 backbone genes have been highly conservative for the last 10 years. This suggests that the IncX3 plasmid backbone gene itself has the advantage of mediating the wide spread of *bla*_*NDM*_. The theory of plasmid biology shows that high conjugation efficiency, powerful distribution system, and low fitness cost are three factors to ensure the long-term existence and wide spread of plasmids (Zwanzig, 2021). The T4SS system in the backbone region guarantees the efficiency of plasmid self-conjugation (Costa et al., 2020); the *parAB* distribution system ensures that progeny plasmids can be assigned to the offspring host, promoting plasmid stability (Sheng et al., 2020). Further research into backbone gene function will facilitate the deep investigation of IncX3 plasmid fitness mechanisms in China.

## Conclusion

In this study, our data indicate that carbapenem-resistant gene-carrying IncX3 plasmids tend to be specifically associated with hospital-isolated *E. coli* ST410 from phylogroup A. Although the backbones of IncX3 plasmids remained highly conservative over the last 10 years, the *bla*_*NDM*_ resistance genetic contexts on plasmid could fall into five subtypes, among which AR_N1_I has been identified in *Enterobacter cloacae174* chromosome and AR_N5_I was simultaneously located on IncF and IncA/C plasmids. This suggests that the *bla*_*NDM*_ resistance gene environment can spread between different plasmids, between different bacterial genera, or between strains and plasmids, highlighting that it is imperative to adopt more stringent infection control measures targeting IncX3 plasmid spread.

## Data Availability Statement

The datasets presented in this study can be found in online repositories. The names of the repository/repositories and accession number(s) can be found in the article/Supplementary Material.

## Author Contributions

CZ, BL, and YG conceived and designed the experiments and wrote the manuscript. NL, JW, FL, and LY searched PubMed and collected IncX3 plasmid information. BL and YG performed the bioinformatic analysis. All authors provide critical input to the manuscript and endorsed the final version.

## Conflict of Interest

The authors declare that the research was conducted in the absence of any commercial or financial relationships that could be construed as a potential conflict of interest.

## Publisher’s Note

All claims expressed in this article are solely those of the authors and do not necessarily represent those of their affiliated organizations, or those of the publisher, the editors and the reviewers. Any product that may be evaluated in this article, or claim that may be made by its manufacturer, is not guaranteed or endorsed by the publisher.
