# Supplementary material for: In silico Evolution and Comparative Genomic Analysis of IncX3 Plasmids Isolated From China Over Ten Years
Source: Front Microbiol. 2021 Dec 3;12:725391. doi: 10.3389/fmicb.2021.725391 (PMC8681339; doi:10.3389/fmicb.2021.725391)
Supplement: Supplementary file 1 [file Data_Sheet_1.docx]

Table S1 **Summary of IncX3 Plasmid Information Collected and Reported in China**

| Number | name | species | city | year | Specimen source | accession number | Ref |
| --- | --- | --- | --- | --- | --- | --- | --- |
| 1 | pNDM5_025943 | ***E.coli*** | Sichuan | 2017 | hospital sewage | CP027204 | (1) |
| 2 | pVH1 | ***E.coli*** | Shandong | 2017 | cucumber | CP028705 | (2) |
| 3 | pNDM-20 | ***E.coli*** | Shandong | 2016 | faecal swab swine | MF458176 | (3) |
| 4 | pAD-19R | ***E.coli*** | Shandong | 2015 | faecal swab chicken | KX833071 | (4) |
| 5 | pNDM-QD29 | ***E.coli*** | Shandong | 2013 | patient urine | KU167609 | (5) |
| 6 | pNDM-QD28 | ***E.coli*** | Shandong | 2013 | patient sputum | KU167608 | (5) |
| 7 | p505108-NDM | ***C.sakazakii*** | Jiangsu | 2016 | patient sputum | KY978629 | (6) |
| 8 | pGZ2-NDM | ***E.coli*** | Guangdong | 2015 | patient abdominal drainage | CM007910 | (7) |
| 9 | p112298-NDM | ***C.freundii*** | Guangdong | 2013 | patient urine | KP987216 | (8) |
| 10 | pP10159-1 | ***C.freundii*** | Chongqing | 2013 | patient urine | MF072961 | (9) |
| 11 | pGB788-IMP | ***E.coli*** | Guangdong | 2016 | healthy human faeces | no | (10) |
| 12 | pNDM13-DC33 | ***E.coli*** | Zhejiang | 2014 | patient urine | KX094555 | (11) |
| 13 | pEC21-OXA-181 | ***E.coli*** | Henan | 2013 | patient blood | MG893567 | (12) |
| 14 | pYQ13500-NDM | *E.cloacae* | Zhejiang | 2013 | patient blood | KR059865 | (13) |
| 15 | pYQ13450 | ***K.pneumoniae*** | Zhejiang | 2013 | patient blood | no | (13) |
| 16 | pNDM1_EC8 | ***E.coli*** | Sichuan | 2012 | - | no^a^ | (14) |
| 17 | pEn_NDM | *E.cloacae* | Zhejiang | 2013 | patient sputum | MH061381 | (15) |
| 18 | pNDM-BJ03 | ***E.cloacae*** | Beijing | 2012 | patient sputum | MF415608 | (16) |
| 19 | pECNDM101 | ***E.coli*** | Sichuan | 2015 | faecal swabs swine | KX507346 | (17) |
| 20 | pJN05NDM-7 | ***E.coli*** | Shandong | 2015 | patient urine | MH523639 | (18) |
| 21 | pOXA181_EC14828 | ***E.coli*** | Sichuan | 2014 | patient blood | KP400525. | (19) |
| 22 | p24835-NDM5 | ***K.pneumoniae*** | Jiangxi | 2015 | patient urine | CP014006 | (20) |
| 23 | pNDM5-LDR | ***K.pneumoniae*** | Zhejiang | 2017 | patient sputum | MK308632 | (21) |
| 24 | pSCM96-2 | ***K. pneumoniae*** | Sichuan | 2017 | patient sputum | CP028718 | (22) |
| 25 | pNDM5_IncX3 | ***K.pneumoniae*** | Jiangsu | 2015 | patient Peritoneal fluid | KU761328 | (23) |
| 26 | pKOX3-P3-NDM | ***Klebsiella oxytoca*** | Shaanxi | 2013 | patient urine | KY913899 | (24) |
| 27 | pSCKLB-2 | ***K.pneumoniae*** | - | 2016 | faecal swab chicken | MH161191 | (25) |
| 28 | pNDM-SX04 | ***K.pneumoniae*** | Shaanxi | 2012 | patient sputum | KC876051 | (26) |
| 29 | pNDM-HK3871-1 | ***E.coli*** | Hong Kong | 2016 | healthy human or patient | MH234497 | (27) |
| 30 | pNDM-HK3855-1 | ***E.coli*** | Hong Kong | 2016 | healthy human or patient | MH234498 | (27) |
| 31 | pNDM-HK3836-1 | ***E.coli*** | Hong Kong | 2016 | healthy human or patient | MH234499 | (27) |
| 32 | pNDM-HK3819-1 | K.pneumoniae | Hong Kong | 2016 | healthy human or patient | MH234500 | (27) |
| 33 | pNDM-HK3816-1 | K.pneumoniae | Hong Kong | 2016 | healthy human or patient | MH234501 | (27) |
| 34 | pNDM-HK3774 | ***E.coli*** | Hong Kong | 2016 | healthy human or patient | MH234502 | (27) |
| 35 | pNDM-HK3712-1 | ***E.coli*** | Hong Kong | 2016 | healthy human or patient | MH234503 | (27) |
| 36 | pNDM-HK3706-1 | K.pneumoniae | Hong Kong | 2016 | healthy human or patient | MH234504 | (27) |
| 37 | pNDM-HK3694 | ***E.coli*** | Hong Kong | 2016 | healthy human or patient | MH234505 | (27) |
| 38 | pNDM-HK3473-1 | E.cloacae | Hong Kong | 2016 | healthy human or patient | MH234506 | (27) |
| 39 | pNDM-HK3218-1 | ***E.coli*** | Hong Kong | 2016 | healthy human or patient | MH234507 | (27) |
| 40 | pNDM-HK2998-1 | K.pneumoniae | Hong Kong | 2016 | healthy human or patient | MH234508 | (27) |
| 41 | pNDM-HK2967 | E.coli | Hong Kong | 2016 | healthy human or patient | MH234509 | (27) |
| 42 | pRJA274 | Raoultella planticola | Shanghai | 2012 | patient drain | KF877335 | (28) |
| 43 | pNDM21_020023 | ***E.coli*** | Sichuan | 2016 | patient urine | CP025948 | (29) |
| 44 | pCQ02-121 | ***E.coli*** | Chongqing | 2015 | rectal swab cat | KU647721 | (30) |
| 45 | pNDM1_CF65 | ***C. freundii*** | Sichuan | 2015 | hospital sewage | no^a^ | (31) |
| 46 | pNDM-HF727 | E. cloacae | Guangdong | 2012 | patient urine | no^a^ | (32) |
| 47 | pNDM5_IncX3 | ***K.pneumoniae*** | Jiangsu | 2015 | patient Surgery wound | KU761328 | (33) |
| 48 | HZW25 p4 | ***K.pneumoniae*** | Zhejiang | 2017 | patient bile | CP025215 | (34) |
| 49 | pNDM5-GZEC065 | ***E.coli*** | Guangdong | 2017 | patient blood | CP048028 | (35) |
| 50 | pGDQ8D112M-NDM | ***K. pneumoniae*** | Guangdong | 2018 | faecal duck | MK628734 | (36) |
| 51 | pGDQ8D117M-NDM | ***K. quasipneumoniae*** | Guangdong | 2018 | faecal duck | MK618661 | (36) |
| 52 | BJS-81 p3 | ***K. pneumoniae*** | Beijing | 2018 | subway environment | MN847777 | (37) |
| 53 | pNDM 5_SH160 | ***S. Typhimurium*** | Shanghai | 2016 | minced pork meat | no^b^ | (38) |
| 54 | pBSI034-NDM1 | ***E. cloacae*** | Guangdong | 2013 | patient blood | MN937240 | (39) |
| 55 | pKP709-OXA-181. | ***K. pneumoniae*** | Zhejiang | 2019 | healthy human faeces | MN227183 | (40) |
| 56 | MH9BT | ***E.coli*** | Shandong | 2018 | faecal horses | MT012582 | (41) |
| 57 | MH10T | ***E.coli*** | Shandong | 2018 | faecal horses | MT012583 | (41) |
| 58 | MH14T | ***E.coli*** | Shandong | 2018-2020 | faecal horses | MT012584 | (41) |
| 59 | MH19T | ***E.coli*** | Shandong | 2018-2020 | faecal horses | MT012585 | (41) |
| 60 | pR15_NDM-5 | ***E.coli*** | Zhejiang | 2017 | Wastewater treatment plants | MK256964 | (42) |
| 61 | pNDM5-GZ04_A | ***E.coli*** | Guangdong | 2018 | patient faeces | CP042339 | (43) |
| 62 | pWLK-NDM | ***R.ornithinolytica*** | Henan | 2018 | urban river systems | CP038280 | (44) |
| 63 | pNDM-K725 | ***K. pneumoniae*** | Shanghai | 2017/3/15 | patient sputum | MK450348 | (45) |
| 64 | pNDM-CR33 | K. aerogenes | Shanghai | 2017 | patient blood | MK450349 | (45) |
| 65 | pNDM-Z214 | ***E.coli*** | Shanghai | 2017 | patient blood | MK450347 | (45) |
| 66 | pNDM-Z244 | ***E.coli*** | Shanghai | 2017 | patient urine | MK450346 | (45) |
| 67 | pNDM-KP14003 | ***K. pneumoniae*** | Beijing | 2014 | patient blood | CP041938 | (46) |
| 68 | pNDM-5, | ***Proteus mirabilis*** | Zhejiang | 2018 | patient urine | CP043333 | (47) |
| 69 | pMER690T plasmid | ***E.coli*** | Shandong | 2017-2018 | leaf rape | no | (48) |
| 70 | pYZPW131 | ***S.Typhimurium*** | Jiangsu | - | retail pork market | MK848866 | (49) |
| 71 | pSL131_ IncX3 | ***Salmonella Lomita*** | Zhejiang | 2015 | patient faeces | MH105050 | (50) |
| 72 | pNDM-CREC-8 | ***E.coli*** | Jiangsu | 2017 | Urine | no | (51) |
| 73 | pL37-3 | ***E.coli*** | Jiangsu | 2018 | swab samples goose | CP034591 | (52) |
| 74 | pL41-1-4 | E.coli | Jiangsu | 2018 | Feed goose | CP034730 | (52) |
| 75 | pL53-4 | ***E.coli*** | Jiangsu | 2018 | water goose | CP034757 | (52) |
| 76 | pL65-9 | E.coli | Jiangsu | 2018 | feces goose | CP034744 | (52) |
| 77 | pL100-4 | ***E.coli*** | Jiangsu | 2018 | anal swab of goose | CP034748 | (52) |
| 78 | pL103-2-5 | E.coli | Jiangsu | 2018 | anal swab of goose | CP034847 | (52) |
| 79 | pNDM1_SCW13 | ***Kluyvera Cryocrescens*** | Sichuan | 2019 | hospital sewage | MN178638 | (53) |
| 80 | pNDM5-SCNJ1 | *K. pneumoniae* | Sichuan | 2018 | patient sputum | MK715437 | (54) |
| 81 | pEC25_NDM-7 | *E.coli* | Zhejiang | 2017 | patient urine | CP035125 | (55) |
| 82 | pNDM5-L241 | *M. morganii* | Zhejiang | 2016 | stool | CP033057 | (56) |
| 83 | pTB203 | *E.coli* | Zhejiang | 2017 | a layer hen farm | CP029245 | (57) |
| 84 | pNDM-HN380 | *K. pneumoniae* | Hunan | 2011 | Patient faeces | JX104760 | (32) |
| a, Plasmid sequence show >99% identity to pNDM-HN380; b, Plasmid sequence show >99% identity to pNDM_MGR194(58) | | | | | | |  |

Table S2 **Using pNDM-HN380 as the reference plasmid, the summary table of IncX3 plasmids with altered backbone genes**

| Num. | Name | positon | type | REF | ALT | NT_POS | AA_POS | EFFECT | Backbone genes |
| --- | --- | --- | --- | --- | --- | --- | --- | --- | --- |
| 1 | pNDM5_025943 | 33310 | snp | T | G | 227/495 | 76/164 | missense_variant c.227A>C p.Gln76Pro | parB |
| 2 | pAD-19R | 39487 | snp | A | T | 458/930 | 153/309 | missense_variant c.458T>A p.Ile153Asn | pilX9 |
|  | pAD-19R | 39685 | snp | A | G | 260/930 | 87/309 | missense_variant c.260T>C p.Leu87Pro | pilX9 |
|  | pAD-19R | 47565 | snp | A | T | 2/651 | 1/216 | start_lost c.2T>A p.Met1? | actX |
| 3 | pNDM-QD29 | 33311 | snp | G | T | 226/495 | 76/164 | missense_variant c.226C>A p.Gln76Lys | parB |
| 4 | pNDM-QD28 | 47183 | snp | G | A | 384/651 | 128/216 | synonymous_variant c.384C>T p.Ile128Ile | actX |
| 5 | pEC21-OXA-181 | 41318 | snp | C | T | 611/1056 | 204/351 | missense_variant c.611G>A p.Cys204Tyr | pilX6 |
| 6 | pECNDM101 | 1972 | snp | C | T | 388/423 | 130/140 | synonymous_variant c.388C>T p.Leu130Leu | taxD |
|  | pECNDM101 | 31302 | snp | A | G | 392/2328 | 131/775 | missense_variant c.392T>C p.Leu131Pro | topB |
|  | pECNDM101 | 41798 | ins | T | TA | 130/1056 | 44/351 | frameshift_variant c.130dupT p.Tyr44fs | pilX6 |
|  | pECNDM101 | 46138 | complex | TGT | GGG | 546/645 | 182/214 | missense_variant c.544_546delACAinsCCC p.Thr182Pro | pilX1 |
| 7 | pOXA181_EC14828 | 41318 | snp | C | T | 611/1056 | 204/351 | missense_variant c.611G>A p.Cys204Tyr | pilX6 |
| 8 | p24835-NDM5 | 42759 | snp | A | C | 219/771 | 73/256 | missense_variant c.219T>G p.Asp73Glu | pilX5 |
| 9 | pSCM96-2 | 33104 | snp | G | A | 433/495 | 145/164 | missense_variant c.433C>T p.Arg145Cys | parB |
| 10 | pKOX3-P3-NDM | 33338 | ins | G | GT | 198/495 | 66/164 | frameshift_variant c.198dupA p.Gln67fs | parB |
|  | pKOX3-P3-NDM | 40206 | ins | G | GT | 472/729 | 158/242 | frameshift_variant c.472dupA p.Thr158fs | pilX8 |
|  | pKOX3-P3-NDM | 40651 | ins | G | GT | 27/729 | 9/242 | frameshift_variant c.27dupA p.Leu10fs | pilX8 |
|  | pKOX3-P3-NDM | 40908 | ins | C | CA | 1020/1056 | 340/351 | frameshift_variant c.1020dupT p.Ala341fs | pilX6 |
|  | pKOX3-P3-NDM | 40974 | ins | A | AT | 954/1056 | 318/351 | frameshift_variant c.954dupA p.Ser319fs | pilX6 |
|  | pKOX3-P3-NDM | 41041 | ins | A | AT | 887/1056 | 296/351 | frameshift_variant c.887dupA p.Asn296fs | pilX6 |
|  | pKOX3-P3-NDM | 46186 | ins | G | GT | 497/645 | 166/214 | frameshift_variant c.497dupA p.Asn166fs | pilX1 |
| 11 | pSCKLB-2 | 42935 | snp | C | A | 43/771 | 15/256 | missense_variant c.43G>T p.Val15Phe | pilX5 |
| 12 | pNDM-HK3712-1 | 35261 | snp | A | G | 1311/1836 | 437/611 | synonymous_variant c.1311T>C p.Gly437Gly | taxB |
| 13 | pNDM-HK2967 | 45681 | snp | C | T | 60/2754 | 20/917 | synonymous_variant c.60G>A p.Pro20Pro | pilX3-pilX4 |
| 14 | pRJA274 | 40523 | del | AC | A | 155/729 | 52/242 | frameshift_variant c.155delG p.Gly52fs | pilX8 |
| 15 | pNDM21_020023 | 1738 | snp | C | T | 154/423 | 52/140 | missense_variant c.154C>T p.His52Tyr | taxD |
| 16 | HZW25 p4 | 37315 | del | CG | C | 293/1035 | 98/344 | frameshift_variant c.293delC p.Pro98fs | pilX11 |
| 17 | pBSI034-NDM1 | 34041 | snp | T | A | 219/315 | 73/104 | synonymous_variant c.219A>T p.Ala73Ala | kikA |
| 18 | pKP709-OXA-181. | 41318 | snp | C | T | 611/1056 | 204/351 | missense_variant c.611G>A p.Cys204Tyr | pilX6 |
|  | pKP709-OXA-181. | 1784 | snp | G | A | 200/423 | 67/140 | missense_variant c.200G>A p.Ser67Asn | taxD |
| 19 | pEC25_NDM-7 | 33104 | snp | G | A | 433/495 | 145/164 | missense_variant c.433C>T p.Arg145Cys | parB |
| 20 | pNDM5-L241 | 478 | snp | C | T | 478/1014 | 160/337 | missense_variant c.478C>T p.Arg160Cys | repB |
| 21 | pTB203 | 52645 | snp | G | A | 39/516 | 13/171 | synonymous_variant c.39C>T p.Ser13Ser | dnaJ |
|  | pTB203 | 3381 | snp | C | A | 531/663 | 177/220 | synonymous_variant c.531G>T p.Arg177Arg | parA |

1. Long H, Feng Y, Ma K, Liu L, McNally A, Zong Z. 2019. The co-transfer of plasmid-borne colistin-resistant genes mcr-1 and mcr-3.5, the carbapenemase gene bla(NDM-5) and the 16S methylase gene rmtB from Escherichia coli. Sci Rep 9:696.

2. Liu BT, Zhang XY, Wan SW, Hao JJ, Jiang RD, Song FJ. 2018. Characteristics of Carbapenem-Resistant Enterobacteriaceae in Ready-to-Eat Vegetables in China. Front Microbiol 9:1147.

3. Liu Z, Li J, Wang X, Liu D, Ke Y, Wang Y, Shen J. 2018. Novel Variant of New Delhi Metallo-β-lactamase, NDM-20, in Escherichia coli. Front Microbiol 9:248.

4. Liu Z, Wang Y, Walsh TR, Liu D, Shen Z, Zhang R, Yin W, Yao H, Li J, Shen J. 2017. Plasmid-Mediated Novel bla(NDM-17) Gene Encoding a Carbapenemase with Enhanced Activity in a Sequence Type 48 Escherichia coli Strain. Antimicrob Agents Chemother 61.

5. Zhu YQ, Zhao JY, Xu C, Zhao H, Jia N, Li YN. 2016. Identification of an NDM-5-producing Escherichia coli Sequence Type 167 in a Neonatal Patient in China. Sci Rep 6:29934.

6. Shi L, Liang Q, Zhan Z, Feng J, Zhao Y, Chen Y, Huang M, Tong Y, Wu W, Chen W, Li X, Yin Z, Wang J, Zhou D. 2018. Co-occurrence of 3 different resistance plasmids in a multi-drug resistant Cronobacter sakazakii isolate causing neonatal infections. Virulence 9:110-120.

7. Zhang Y, Liao K, Gao H, Wang Q, Wang X, Li H, Wang R, Wang H. 2017. Decreased Fitness and Virulence in ST10 Escherichia coli Harboring bla(NDM-5) and mcr-1 against a ST4981 Strain with bla(NDM-5). Front Cell Infect Microbiol 7:242.

8. Feng J, Qiu Y, Yin Z, Chen W, Yang H, Yang W, Wang J, Gao Y, Zhou D. 2015. Coexistence of a novel KPC-2-encoding MDR plasmid and an NDM-1-encoding pNDM-HN380-like plasmid in a clinical isolate of Citrobacter freundii. J Antimicrob Chemother 70:2987-91.

9. Ouyang J, Sun F, Zhou D, Feng J, Zhan Z, Xiong Z, Yang B, Liu Z, Li T, Tong Y, Xia P. 2018. Comparative genomics of five different resistance plasmids coexisting in a clinical multi-drug resistant Citrobacter freundii isolate. Infect Drug Resist 11:1447-1460.

10. Feng S, Shen C, Chen H, Zheng X, Xia Y, Zhong LL, Huang X, Wu X, Tian GB. 2018. Co-production of MCR-1 and NDM-5 in Escherichia coli isolated from a colonization case of inpatient. Infect Drug Resist 11:1157-1161.

11. Lv J, Qi X, Zhang D, Zheng Z, Chen Y, Guo Y, Wang S, Chen L, Kreiswirth BN, Tang YW, Chen Z, Hu L, Wang L, Yu F. 2016. First Report of Complete Sequence of a bla(NDM-13)-Harboring Plasmid from an Escherichia coli ST5138 Clinical Isolate. Front Cell Infect Microbiol 6:130.

12. Qin S, Cheng J, Wang P, Feng X, Liu HM. 2018. Early emergence of OXA-181-producing Escherichia coli ST410 in China. J Glob Antimicrob Resist 15:215-218.

13. Yang Q, Fang L, Fu Y, Du X, Shen Y, Yu Y. 2015. Dissemination of NDM-1-Producing Enterobacteriaceae Mediated by the IncX3-Type Plasmid. PLoS One 10:e0129454.

14. Feng Y, Yang P, Xie Y, Wang X, McNally A, Zong Z. 2015. Escherichia coli of sequence type 3835 carrying bla NDM-1, bla CTX-M-15, bla CMY-42 and bla SHV-12. Sci Rep 5:12275.

15. Chavda B, Lv J, Hou M, Chavda KD, Kreiswirth BN, Feng Y. 2018. Coidentification of mcr-4.3 and bla(NDM-1) in a Clinical Enterobacter cloacae Isolate from China. 62.

16. Lü Y, Liu W, Liang H, Zhao S, Zhang W, Liu J, Jin C, Hu H. 2018. NDM-1 encoded by a pNDM-HN380-like plasmid pNDM-BJ03 in clinical Enterobacter cloacae. Diagn Microbiol Infect Dis 90:153-155.

17. Kong LH, Lei CW, Ma SZ, Jiang W, Liu BH, Wang YX, Guan R, Men S, Yuan QW, Cheng GY, Zhou WC, Wang HN. 2017. Various Sequence Types of Escherichia coli Isolates Coharboring blaNDM-5 and mcr-1 Genes from a Commercial Swine Farm in China. Antimicrob Agents Chemother 61.

18. Hao Y, Shao C, Bai Y, Jin Y. 2018. Genotypic and Phenotypic Characterization of IncX3 Plasmid Carrying bla (NDM-7) in Escherichia coli Sequence Type 167 Isolated From a Patient With Urinary Tract Infection. Front Microbiol 9:2468.

19. Liu Y, Feng Y, Wu W, Xie Y, Wang X, Zhang X, Chen X, Zong Z. 2015. First Report of OXA-181-Producing Escherichia coli in China and Characterization of the Isolate Using Whole-Genome Sequencing. Antimicrob Agents Chemother 59:5022-5.

20. Mei YF, Liu PP, Wan LG, Liu Y, Wang LH, Wei DD, Deng Q, Cao XW. 2017. Virulence and Genomic Feature of a Virulent Klebsiella pneumoniae Sequence Type 14 Strain of Serotype K2 Harboring bla(NDM-5) in China. Front Microbiol 8:335.

21. Wang Z, Li M, Shen X, Wang L, Liu L, Hao Z, Duan J, Yu F. 2019. Outbreak of bla(NDM-5)-Harboring Klebsiella pneumoniae ST290 in a Tertiary Hospital in China. Microb Drug Resist 25:1443-1448.

22. Liu Y, Zhang H, Zhang X, Jiang N, Zhang Z, Zhang J, Zhu B, Wang G, Zhao K, Zhou Y. 2019. Characterization of an NDM-19-producing Klebsiella pneumoniae strain harboring 2 resistance plasmids from China. Diagn Microbiol Infect Dis 93:355-361.

23. Du H, Chen L, Tang Y-W, Kreiswirth BN. 2016. Emergence of the mcr-1 colistin resistance gene in carbapenem-resistant Enterobacteriaceae. The Lancet Infectious Diseases 16:287-288.

24. Wang J, Yuan M, Chen H, Chen X, Jia Y, Zhu X, Bai L, Bai X, Fanning S, Lu J, Li J. 2017. First Report of Klebsiella oxytoca Strain Simultaneously Producing NDM-1, IMP-4, and KPC-2 Carbapenemases. Antimicrob Agents Chemother 61.

25. Xiang R, Zhang AY, Ye XL, Kang ZZ, Lei CW, Wang HN. 2018. Various Sequence Types of Enterobacteriaceae Isolated from Commercial Chicken Farms in China and Carrying the bla(NDM-5) Gene. Antimicrob Agents Chemother 62.

26. Wang X, Xu X, Li Z, Chen H, Wang Q, Yang P, Zhao C, Ni M, Wang H. 2014. An outbreak of a nosocomial NDM-1-producing Klebsiella pneumoniae ST147 at a teaching hospital in mainland China. Microb Drug Resist 20:144-9.

27. Wang Y, Tong M-K, Chow K-H, Cheng VC-C, Tse CW-S, Wu AK-L, Lai RW-M, Luk W-K, Tsang DN-C, Ho P-L. 2018. Occurrence of Highly Conjugative IncX3 Epidemic Plasmid Carrying blaNDM in Enterobacteriaceae Isolates in Geographically Widespread Areas. Frontiers in Microbiology 9:2272.

28. Qu H, Wang X, Ni Y, Liu J, Tan R, Huang J, Li L, Sun J. 2015. NDM-1-producing Enterobacteriaceae in a teaching hospital in Shanghai, China: IncX3-type plasmids may contribute to the dissemination of blaNDM-1. Int J Infect Dis 34:8-13.

29. Hammerum AM, Hasman H, Liu L, Feng Y, McNally A, Zong Z. 2018. blaNDM-21, a new variant of blaNDM in an Escherichia coli clinical isolate carrying blaCTX-M-55 and rmtB. mSphere 73:2336-2339.

30. Sun J, Yang RS, Zhang Q, Feng Y, Fang LX, Xia J, Li L, Lv XY, Duan JH, Liao XP, Liu YH. 2016. Co-transfer of bla(NDM-5) and mcr-1 by an IncX3-X4 hybrid plasmid in Escherichia coli. Nat Microbiol 1:16176.

31. Wu W, Espedido B, Feng Y, Zong Z. 2016. Citrobacter freundii carrying blaKPC-2 and blaNDM-1: characterization by whole genome sequencing. Sci Rep 6:30670.

32. Ho PL, Li Z, Lo WU, Cheung YY, Lin CH, Sham PC, Cheng VC, Ng TK, Que TL, Chow KH. 2012. Identification and characterization of a novel incompatibility group X3 plasmid carrying bla NDM-1 in Enterobacteriaceae isolates with epidemiological links to multiple geographical areas in China. Emerg Microbes Infect 1:e39.

33. Li A, Yang Y, Miao M, Chavda KD, Mediavilla JR, Xie X, Feng P, Tang YW, Kreiswirth BN, Chen L, Du H. 2016. Complete Sequences of mcr-1-Harboring Plasmids from Extended-Spectrum-β-Lactamase- and Carbapenemase-Producing Enterobacteriaceae. Antimicrob Agents Chemother 60:4351-4.

34. Chen Q, Zhou J, Wu S, Yang Y, Yu D, Wang X, Wu M. 2020. Characterization of the IncX3 Plasmid Producing bla (NDM-7) From Klebsiella pneumoniae ST34. Front Microbiol 11:1885.

35. Lin Y, Yang L, Lu L, Wang K, Li J, Li P, Liu Y, Liu X, Li P, Song H. 2020. Genomic features of an Escherichia coli ST156 strain harboring chromosome-located mcr-1 and plasmid-mediated bla(NDM-5). Infect Genet Evol 85:104499.

36. Ma Z, Liu J, Yang J, Zhang X, Chen L, Xiong W, Zeng Z. 2020. Emergence of bla(NDM)-carrying IncX3 plasmid in Klebsiella pneumoniae and Klebsiella quasipneumoniae from duck farms in Guangdong Province, China. J Glob Antimicrob Resist 22:703-705.

37. Cao T, Liu Y, Li Y, Wang Y, Shen Z, Shao B, Walsh TR, Shen J, Wang S. 2020. A public health concern: emergence of carbapenem-resistant Klebsiella pneumoniae in a public transportation environment. J Antimicrob Chemother 75:2769-2772.

38. Gao Y, Wen J, Wang S, Xu X, Zhan Z, Chen Z, Bai J, Qu X, Zhang H, Zhang J, Liao M. 2020. Plasmid-Encoded bla(NDM-5) Gene That Confers High-Level Carbapenem Resistance in Salmonella Typhimurium of Pork Origin. Infect Drug Resist 13:1485-1490.

39. Lin M, Yang Y, Yang Y, Chen G, He R, Wu Y, Zhong LL, El-Sayed Ahmed MAE, Feng S, Shen C, Wen X, Huang J, Li H, Zheng X, Tian GB. 2020. Co-Occurrence of mcr-9 and bla (NDM-1) in Enterobacter cloacae Isolated from a Patient with Bloodstream Infection. Infect Drug Resist 13:1397-1402.

40. Liu C, Fang Y, Zeng Y, Lu J, Sun Q, Zhou H, Shen Z, Chen G. 2020. First Report of OXA-181-Producing Klebsiella pneumoniae in China. Infect Drug Resist 13:995-998.

41. Wang H, Li X, Liu BT. 2020. Occurrence and characterization of KPC-2-producing ST11 Klebsiella pneumoniae isolate and NDM-5-producing Escherichia coli isolate from the same horse of equestrian clubs in China. Transbound Emerg Dis doi:10.1111/tbed.13614.

42. Han H, Liu W, Cui X, Cheng X, Jiang X. 2020. Co-Existence of mcr-1 and bla (NDM-5) in an Escherichia coli Strain Isolated from the Pharmaceutical Industry, WWTP. Infect Drug Resist 13:851-854.

43. Shen Z, Wang Y, Walsh TR, Shen J, Yang L, Lin Y, Lu L, Xue M, Ma H, Guo X, Wang K, Li P, Du X, Qi K, Li P, Song H. 2020. Coexistence of Two bla (NDM-) (5) Genes Carried on IncX3 and IncFII Plasmids in an Escherichia coli Isolate Revealed by Illumina and Nanopore Sequencing. Microorganisms 11:195.

44. Dang B, Zhang H, Li Z, Ma S, Xu Z. 2020. Coexistence of the bla(NDM-1)-carrying plasmid pWLK-NDM and the bla(KPC-2)-carrying plasmid pWLK-KPC in a Raoultella ornithinolytica isolate. Sci Rep 10:2360.

45. Tian D, Wang B, Zhang H, Pan F, Wang C, Shi Y, Sun Y. 2020. Dissemination of the bla (NDM-5) Gene via IncX3-Type Plasmid among Enterobacteriaceae in Children. mSphere 5.

46. Li J, Hu X, Yang L, Lin Y, Liu Y, Li P, Wang K, Qiu S, Li P, Song H. 2020. New Delhi Metallo-β-Lactamase 1-Producing Klebsiella pneumoniae ST719 Isolated from a Neonate in China. Microb Drug Resist 26:492-496.

47. Sun L, Xu J, He F. 2019. Genomic characterisation of a Proteus mirabilis clinical isolate from China carrying bla(NDM-5) on an IncX3 plasmid. J Glob Antimicrob Resist 19:317-319.

48. Liu BT, Song FJ. 2019. Emergence of two Escherichia coli strains co-harboring mcr-1 and bla (NDM) in fresh vegetables from China. Infect Drug Resist 12:2627-2635.

49. Wang Z, He J, Li Q, Tang Y, Wang J, Pan Z, Chen X, Jiao X. 2020. First Detection of NDM-5-Positive Salmonella enterica Serovar Typhimurium Isolated from Retail Pork in China. Microb Drug Resist 26:434-437.

50. Li R, Xie M, Liu L, Huang Y, Wu X, Wang Z, Chan EWC, Chen S. 2020. Characterisation of a cointegrate plasmid harbouring bla(NDM-1) in a clinical Salmonella Lomita strain. Int J Antimicrob Agents 55:105817.

51. Sun P, Xia W, Liu G, Huang X, Tang C, Liu C, Xu Y, Ni F, Mei Y, Pan S. 2019. Characterization Of bla (NDM-5)-Positive Escherichia coli Prevalent In A University Hospital In Eastern China. Infect Drug Resist 12:3029-3038.

52. Liu Z, Xiao X, Li Y, Liu Y, Li R, Wang Z. 2019. Emergence of IncX3 Plasmid-Harboring bla (NDM-) (5) Dominated by Escherichia coli ST48 in a Goose Farm in Jiangsu, China. Front Microbiol 10:2002.

53. Li Y, Luo L, Xiao Z, Wang G, Li C, Zhang Z, Zhou Y, Zhang L. 2019. Characterization of a Carbapenem-Resistant Kluyvera Cryocrescens Isolate Carrying Blandm-1 from Hospital Sewage. 8.

54. Yuan Y, Li Y, Wang G, Li C, Chang YF, Chen W, Nian S, Mao Y, Zhang J, Zhong F, Zhang L. 2019. bla (NDM-5) carried by a hypervirulent Klebsiella pneumoniae with sequence type 29. Antimicrob Resist Infect Control 8:140.

55. Xu J, He F. 2019. Characterization of a NDM-7 carbapenemase-producing Escherichia coli ST410 clinical strain isolated from a urinary tract infection in China. Infect Drug Resist 12:1555-1564.

56. Guo X, Rao Y, Guo L, Xu H, Lv T, Yu X, Chen Y, Liu N, Han H, Zheng B. 2019. Detection and Genomic Characterization of a Morganella morganii Isolate From China That Produces NDM-5. Front Microbiol 10:1156.

57. Tang B, Chang J, Cao L, Luo Q, Xu H, Lyu W, Qian M, Ji X, Zhang Q, Xia X, Yang H. 2019. Characterization of an NDM-5 carbapenemase-producing Escherichia coli ST156 isolate from a poultry farm in Zhejiang, China. BMC Microbiol 19:82.

58. Krishnaraju M, Kamatchi C, Jha AK, Devasena N, Vennila R, Sumathi G, Vaidyanathan R. 2015. Complete sequencing of an IncX3 plasmid carrying blaNDM-5 allele reveals an early stage in the dissemination of the blaNDM gene. Indian J Med Microbiol 33:30-8.

Figure S1 as below

**
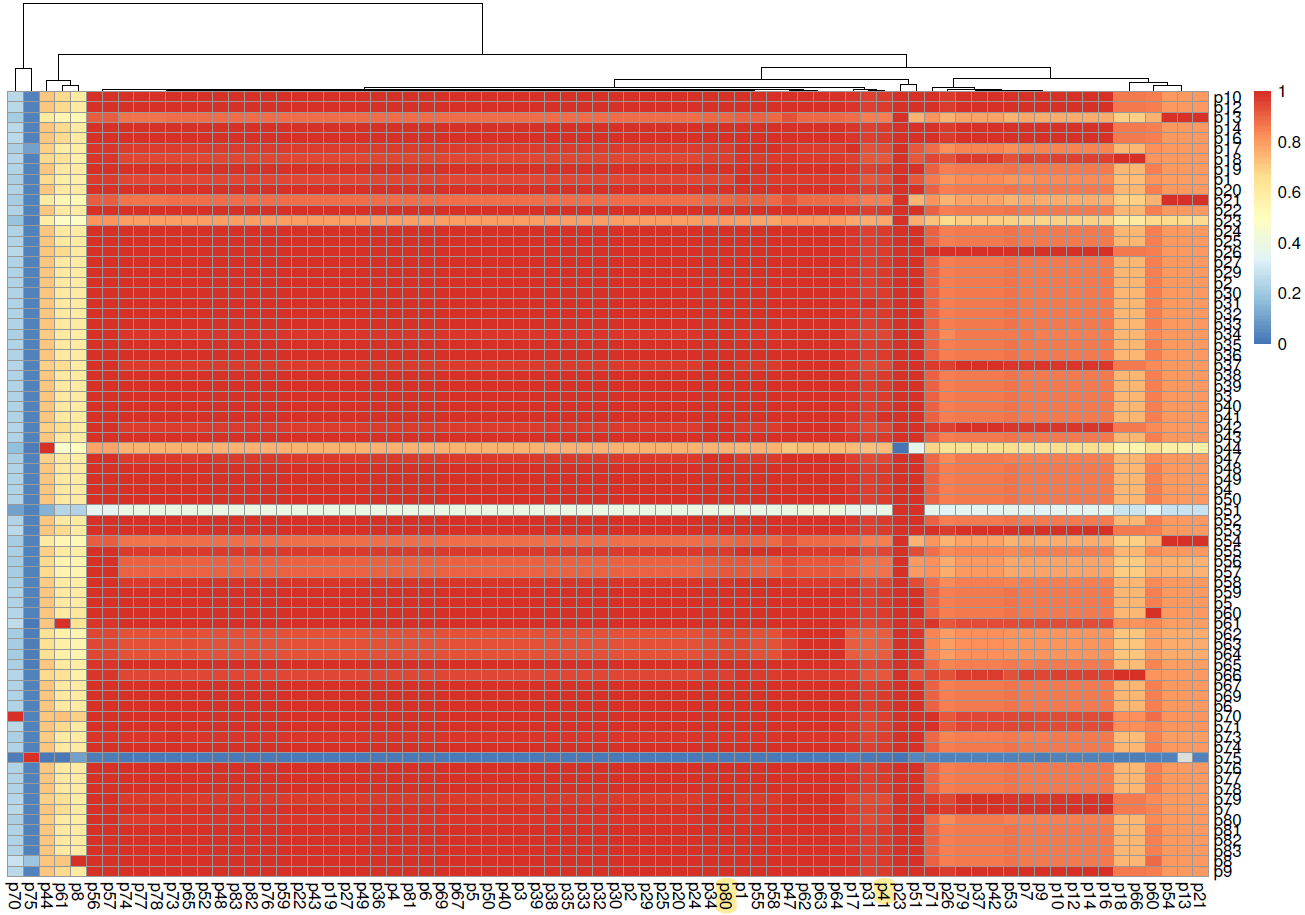
**

Figure S1 Pairwise comparison diagram of 76 whole genome sequences of IncX3 plasmids
